# Artificial intelligence-powered advancements in atrial fibrillation diagnostics: a systematic review

**DOI:** 10.1186/s43044-025-00670-y

**Published:** 2025-07-23

**Authors:** Sofia Khaja, Kevin Baijoo, Reza Aziz

**Affiliations:** City University of New York (CUNY) School of Medicine, New York, United States

**Keywords:** Atrial fibrillation, Artificial intelligence, Arrhythmia diagnostics, Machine learning, Neural networks, Wireless heart monitors, Wearable AI Devices

## Abstract

**Background:**

Cardiovascular diseases remain one of the leading causes of mortality worldwide, with atrial fibrillation emerging as a clinically significant arrhythmia. The increasing prevalence of atrial fibrillation calls for advanced diagnostic tools for accurate detection to reduce adverse consequences, such as stroke and heart failure. Cardiovascular advancements in artificial intelligence have improved the detection and management of atrial fibrillation.

**Objective:**

This review examines recent advancements in atrial fibrillation detection using artificial intelligence-driven tools—such as wearables, neural networks, and machine learning—and highlights their clinical relevance, limitations, and potential to transform cardiovascular care.

**Methodology:**

A systematic review was conducted using PubMed, IEEE Xplore, and ScienceDirect to identify peer-reviewed studies between 2020 and 2024. Original clinical studies using artificial intelligence were included for the diagnosis of atrial fibrillation. Studies on conditions other than atrial fibrillation or incomplete data were excluded. Factors analyzed across all studies included diagnostic application, key findings, clinical implications, and limitations of artificial intelligence approaches.

**Results:**

This review evaluated 11 studies on artificial intelligence-enhanced tools for atrial fibrillation diagnostics. Neural networks showed the highest diagnostic accuracy, outperforming clinicians in retrospective electrocardiogram analyses (80% vs. 75%). Wearable artificial intelligence-integrated devices, such as electrocardiogram wristbands, offer the highest accessibility and real-time monitoring, with sensitivities exceeding 94%, although they are limited by single-lead input and patient compliance. Machine learning models, including random forest and XGBoost, showed moderate performance (AUROC 0.74–0.89) with strengths in risk prediction and stratification. Key challenges included limited generalizability, small-sample sizes, and varying model accuracy.

**Conclusions:**

This review highlights the potential of artificial intelligence to improve atrial fibrillation diagnostics through wearable technologies, neural networks, and machine learning. While these tools often outperform traditional methods, real-world use is limited by small, retrospective studies and a lack of validation. Future work should focus on equity, transparency, and expanding artificial intelligence use beyond atrial fibrillation diagnosis, with collaboration needed to ensure safe, effective clinical integration.

## Background

Cardiovascular diseases (CVDs) are the leading cause of death globally, demanding the need for accurate and timely diagnosis [1]. Among them, atrial fibrillation (AFib) is the most common sustained arrhythmia and a significant risk factor for stroke and heart failure [2], with current estimations of 3.046 million new reported diagnoses worldwide in 2017 [3]. The current worldwide prevalence of AFib is estimated to be 37.5 million cases (0.51% of the worldwide population), with a 33% increase noted over the past 20 years [3]. AFib increases the risk of ischemic stroke by a factor of three to five times, with AFib being responsible for approximately 15% of all strokes worldwide [4]. Early detection of AFib and other cardiac conditions is crucial in improving patient outcomes and reducing healthcare burdens. However, traditional diagnostic methods, such as intermittent ECGs and Holter monitors, often fail to capture paroxysmal or asymptomatic causes and are also limited by human error, observer variability, and numerous time constraints in clinical practice [5]. These challenges have driven a paradigm shift toward integrating artificial intelligence (AI) models, particularly in AFib diagnostics, highlighting the need for more scalable and accurate diagnostics approaches. While prior reviews have discussed AI’s potential in general cardiology and imaging [6–8], few have critically examined its specific clinical performance in AFib detection, particularly using the most recent literature and diverse diagnostic platforms.

Beyond arrhythmias, AI-enhanced echocardiography, cardiac magnetic resonance imaging (MRI), and computed tomography angiography (CTA) offer enhanced image analysis, assisting clinicians in diagnosing structural and functional cardiac abnormalities [6]. Wearable AI devices have also shown promise in helping patients monitor their cardiac rhythms in real-time, allowing for early detection and possible screening of life-threatening arrhythmias, such as AFib and ventricular fibrillation [7]. If implemented in the clinical setting, these modern advancements will enhance diagnostic precision and help reduce socioeconomic disparities that contribute to the higher rate of cardiac abnormalities observed in resource-limited settings [8].

AI models, particularly machine learning (ML) and deep learning (DL), have also demonstrated promising advancements in cardiovascular diagnosis by identifying patterns that are often missed by human observers [9]. Algorithms, such as random forest (RF), support vector machines (SVM), eXtreme gradient boosting (XGB), and logistic regressions, have been applied to risk prediction models and arrhythmia classifications [10].

This systematic review addresses that gap by evaluating current evidence on AI-driven tools for AFib diagnostics. We assess and discuss the diagnostic accuracy, clinical relevance, social relevance, deployment barriers, and limitations of these models, aiming to clarify how AI can address the unmet challenges in AFib detection and guide future implementation in cardiology practice.

## Methods

### Design

A systematic literature review was conducted to examine existing results. A review methodology was used to analyze AI models in AFib diagnostics.

### Search criteria

The following databases were used: PubMed, IEEE Xplore, and ScienceDirect. This search criteria were limited to peer-reviewed articles published from January 2020 to December 2024. The following search string was used: (“atrial fibrillation”[MeSH Terms] OR “AFib” OR “arrhythmia”) AND (“artificial intelligence” OR “machine learning” OR “deep learning” OR “neural networks”) AND (“diagnostics” OR “screening” OR “detection”). Boolean operators (AND/OR) were systematically applied to refine search sensitivity and specificity. Filters for English language, human subjects, and peer-reviewed original research were also used. The last date search was on December 24, 2025.

To assess methodological quality, we applied the QUADAS-2 tool, which evaluates diagnostic accuracy studies across four domains: patient selection, index test, reference standard, and flow and timing. The risk of bias for each study was summarized in a QUADAS-2 table (Additional file 1: Table 1).

**Table 1:** QUADAS-2 Table assessing methodological quality and risk of bias

Study	Patient selection	Index test	Reference standard	Flow and timing	Overall risk of bias
Kumar et al., 2020 [11]	High	Unclear	Low	Unclear	High
Fu et al., 2021 [12]	High	Low	Unclear	Low	High
Huang et al., 2021 [13]	Low	Low	Low	High	High
Zhu et al., 2020 [14]	Low	Low	Low	Low	Low
Cai et al., 2020 [15]	Low	Low	Unclear	Low	Low
Gruwez et al., 2023 [16]	Low	Low	Unclear	Low	Low
Kim et al., 2022 [17]	Low	Low	Unclear	Unclear	Low
Hill et al., 2022 [18]	Low	Unclear	Low	Unclear	Unclear
Gue et al., 2023 [19]	High	Low	Unclear	Unclear	High
Kao et al., 2023 [20]	High	Unclear	Unclear	Low	High
Ma et al., 2023 [21]	High	Unclear	Unclear	Low	High

The methodological quality of the included studies was mixed. Most studies have shown a low risk in the index test and reference standard domains, especially those using standardized ECG readings or validated AI tools. However, several studies had patient selection bias due to small or narrowly defined cohorts, such as elderly-only groups or single-center retrospective designs. Flow and timing were often unclear, with missing details on diagnostic intervals or follow-up. Four studies were rated as having a high risk of bias overall, primarily due to limited external validation, generalizability, and unclear standards for sample selection (Table 1). These limitations were considered in interpreting our review findings.

### Inclusion/exclusion criteria

Studies were selected based on the following criteria:

#### Inclusion criteria

1. Peer-reviewed clinical studies published in English from 2020 to 2024
2. Studies that focused on the use of AI in the management, monitoring, or diagnosis of *atrial fibrillation*
3. RCT and clinical trials only with original data
4. Studies that provided data on the integration of wearable technology into clinical workflows and its impact on healthcare delivery.
5. Studies with full papers available

#### Exclusion criteria

1. Studies that focused on the use of artificial intelligence for conditions other than atrial fibrillation
2. Articles that did not include original research data, such as editorials, commentaries, or opinion pieces.
3. Studies with incomplete data or those not published in peer-reviewed journals
4. Non-English language studies were excluded to maintain consistency in the analysis
5. Exclude all articles published before 2020

#### Data extraction

The following information was collected from each study: study characteristics, AI model or technology, application, study population or dataset, key findings, limitations, and clinical implications. Key outcomes related to atrial fibrillation management and diagnosis were compared to traditional methods. The data was then analyzed to identify the strengths and limitations of different AI models in diagnosing AFib. A qualitative synthesis was conducted to provide a comprehensive overview of the topic.

#### Study selection

Initially, two authors independently screened titles and abstracts relevant to systemic reviews. All three authors reviewed all full-text research articles that met the eligibility criteria until a consensus was reached. Any discrepancies were resolved through discussion with the three reviewers. Any recent primary studies or related reviews were manually screened for additional relevant studies.

**Fig. 1:**
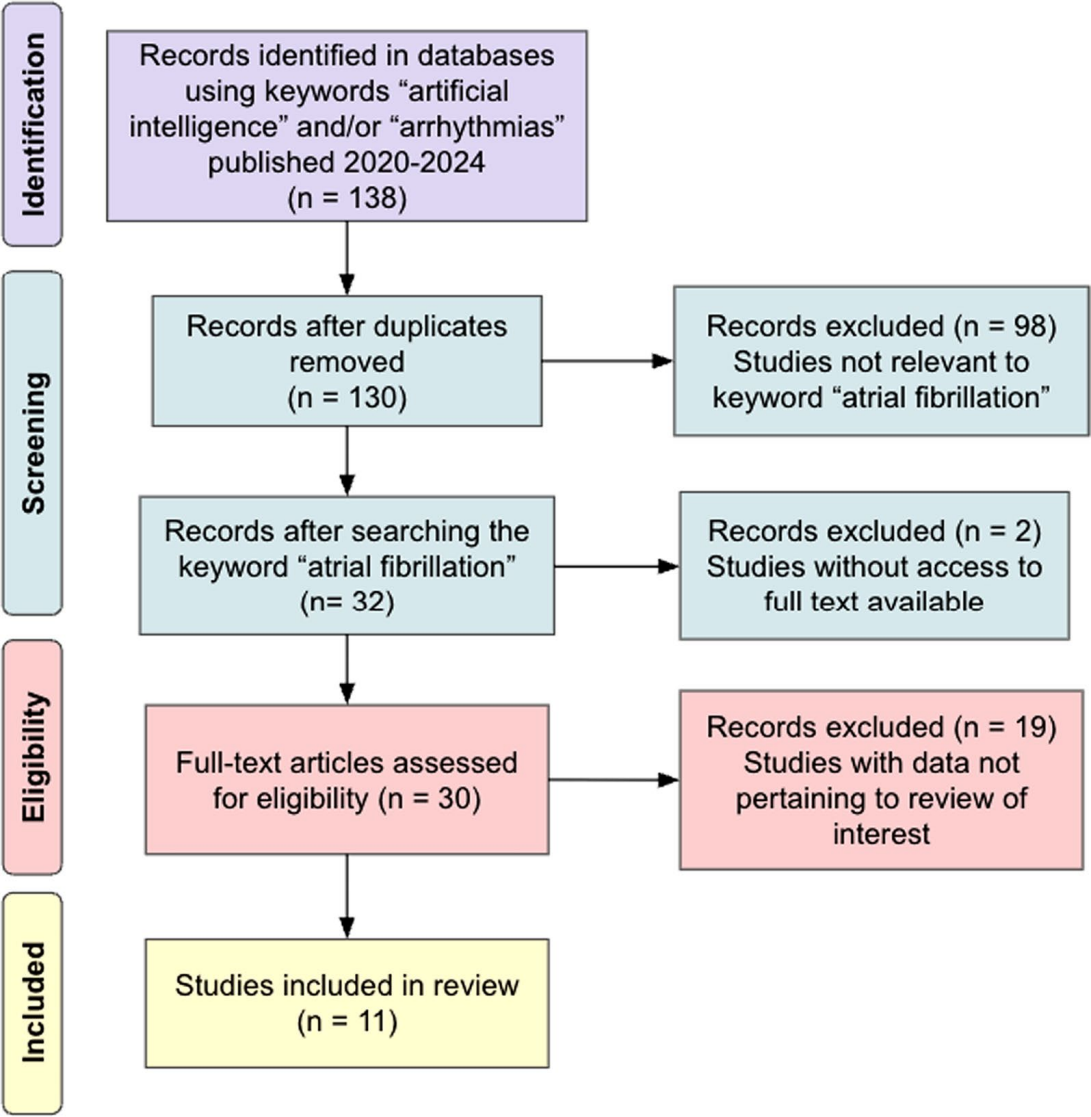
PRISMA flow diagram illustrating study screening process

A total of 138 records were identified through database searches (PubMed, IEEE Xplore, and ScienceDirect). After removing eight duplicate records, 130 articles remained for screening by title and abstract. Of these, 98 were excluded because they did not specifically relate to atrial fibrillation. The full text of 32 records was assessed, with two excluded due to lack of access and 19 excluded for not meeting the inclusion criteria. Ultimately, 11 studies were included in the systematic review. Figure 1 illustrates the PRISMA (Preferred Reporting Items for Systematic Reviews and Meta-Analyses, 2020) flow diagram for this study (Additional file 1: Fig. 1) [22].

## Results

In total, 138 articles were compiled from various databases; however, eight were found to be duplicate articles and were thus removed. Ninety-eight studies were then removed due to relevance specifically to “atrial fibrillation.” Ultimately, 30 documents were identified as potentially relevant, and of these, 19 were excluded following full-text reviews. The remaining 11 studies on different AI models in AFib diagnostics were included and summarized in Table 2 (Additional file 2: Table 2).

**Table 2:** Summary of AI technologies, study population, key findings, limitations, and clinical implications

Study	AI model/technology	Application	Study population/dataset	Key findings	Limitations	Clinical implication
Wearable technologies						
Kumar et al., 2020 [11]	SanketLife wireless ECG biosensor	Wireless ECG biosensor, AI-enhanced ECG data analysis	100 patients, tertiary cardiac care setting, India	High sensitivity (98.15%) and specificity (100%) in diagnosing major cardiovascular conditions	Single-study site, small-sample, challenges detecting time dependent abnormalities	Portable, cost-effective ECG technology suitable for emergencies and outpatient care
Fu et. al., 2021 [12]	Wearable ECG wristband + Mobile AI application	AI algorithm integrated with a dynamic ECG wristband for real-time arrhythmia classification and detection of AF	114 participants (61 with sinus rhythm, 53 with AF), Shanghai Chest Hospital, China	Improved diagnostic accuracy across various postures (97.37%) and sensitivity (94.34%)	Small-sample size, limited to asymptomatic AF, and not designed for detecting other arrhythmias like premature beats or flutter	Accessible wearable dynamic ECG recorder with an AI algorithm can detect AF at different postures, user-friendly and accurate
Huang et al., 2021 [13]	AI algorithm and automated AF detection algorithm	Handheld BigThumb ECG device with AI-enhanced rhythm monitoring recurrence of AF post-ablation	218 patients, randomized into BigThumb group (n = 109) and traditional follow-up group (n = 109), Changhai Hospital, China	Lower AFib recurrence detection rates post-ablation (64.2% vs. 78.9%, *p* = 0.0163) but high diagnostic sensitivity (94.4%) and specificity (98.5%)	Single-center study, small-sample size, limited follow-up period, monitoring compliance varied, with lower usage after three months and at nighttime	Demonstrates AI’s potential for accurate and scalable, cost-effective, noninvasive AF monitoring, reducing undetected episodes
Neural networks						
Zhu et al., 2020 [14]	Deep learning, convolutional neural network	Multi-label diagnosis of heart rhythm or conduction abnormalities	180,112 ECGs from 70,692 patients; test dataset: 828 patients, Wuhan, China	AI model achieved excellence model performance for AF diagnostics (80%), outperforming cardiologists (75%), model had a sensitivity of 0·867 (0·849–0·885) and specificity of 0·995 (0·994–0·996)	Geographic and demographic constraints, lack of certain arrhythmias, rare, complex multi-label ECG cases	Demonstrates AI’s capability for accurate AF detection, reducing diagnostic errors, and supporting decision-making in diverse clinical environments
Cai et al., 2020 [15]	Multi-ECGNet (deep learning: ResNet, SE module, depthwise separable convolution)	Multi-label classification for 55 arrhythmias including AF	32,142 samples (24,106 for training, 8,036 for testing), China	AI model achieved superior AF detection performance compared to cardiologists, supporting clinical decision-making, micro-F1 score of 0.863 for AFib classification—exceeding cardiologists’ performance (0.780)	Dataset imbalance due to underrepresentation of rare arrhythmias; no external clinical validation	Demonstrates the potential for AI to enhance AF detection and reduce diagnostic errors, paving the way for scalable, automated ECG analysis in clinical care
Gruwez et. al., 2023 [16]	Deep neural network (DNN)	Identifying paroxysmal AF from ECGs in sinus rhythm (SR)	494,042 ECGs from 142,310 patients and external validation on 70,172 ECGs from 26,122 patients, Belgium	The existing DNN approach was validated for patients with underlying paroxysmal AF from a 12-lead ECG in sinus rhythm, sensitivity of (95% CI 79.0–82.3%) and specificity (95% CI 79.5–83.4%)	Retrospective design; labeling inconsistencies due to reliance on automated ECG interpretations; limited dataset diversity; prevalence higher than general population	AI-based ECG analysis could aid early detection of latent paroxysmal AF, guiding monitoring and anticoagulation in high-risk patients
Machine learning						
Kim et al., 2022 [17]	Random forest (RF), support vector machine (SVM), eXtreme gradient boosting (XGB)	Prediction of clinically relevant atrial high-rate episodes (AHREs) using patient data before pacemaker implantation	721 patients from 11 hospitals in Korea; divided into 567 for model training and 154 for validation	The ML models demonstrated higher AUROC values than logistic regression (RF: 0.742, SVM: 0.675, XGB: 0.745 vs. LR: 0.669) and better calibration via Brier scores (XGB: 0.021 vs. LR: 0.013)	Small, imbalanced dataset; no progression prediction for clinical AF, stroke, or mortality; results not generalizable to diverse populations	The models predictions help identify patients at high risk of developing AF after pacemaker implantation, enabling targeted interventions to reduce complications like stroke
Hill et. al., 2022 [18]	Machine learning (ML)-based PULsE-AI risk prediction algorithm combined with diagnostic testing	Identifying individuals at high risk of undiagnosed AF in primary care settings, followed by ECG diagnostics	23,745 participants (June 2019–February 2021), six general practices, England	ML-based PULsE-AI identified approximately 45,493 new AFib cases in a high-risk population of 3.3 million, with 50% sensitivity and 90% specificity	Limited generalizability; slow implementation of algorithm; use of a single high-risk threshold (7.4%); resource needs for wider integration not captured	Scalable, targeted screening for AF; reduced primary care burden; earlier identification and treatment of AF; cost-effective approach
Gue et. al., 2023 [19]	Random forest (RF), adaptive boosting (AdaBoost), XGBoost, neural network	Predicting new-onset AF as an adverse event in heart failure patients	2,219 patients from the WARCEF trial from North America, Europe, and South America	AdaBoost achieved the highest performance with an accuracy of 67.0%, F1 score of 0.25, and an AUC of 0.62 (95% CI 0.51–0.73), outperforming XGBoost, random forest, and neural network	Post hoc analysis, limited diversity, potential AF underestimation, and random imputation effects	Emphasizes ML’s role in early AF detection and social factors in risk, urging research on multiracial cohorts and tailored interventions
Kao et al., 2023 [20]	Random forest, decision tree, logistic regression, support vector machine (SVM)	Predicting new-onset AF in older adults for early intervention without ECGs	2,138 AF patients and 8,552 matched controls (age 65–90 years), Taipei Medical University, Taiwan	RF showed higher performance with AUROC of 0.74 and specificity of 98.7%. The AUROC and AUPRC derived from the RF algorithm were 0.74 and 0.89, respectively	Low sensitivity, reliance on ICD-9-CM codes, missing personal data (e.g., lifestyle factors), and lack of temporal effects modeling	Supports precision medicine by identifying high-risk patients for AF screening, optimizing resource use, and reducing unnecessary testing
Ma et al., 2023 [21]	Random forest (RF) algorithm with SHAP analysis	Predicting high-risk paroxysmal AF recurrence after catheter ablation	471 patients (January 2018–December 2020), split into training and testing groups, Air Force Medical University, China	AdaBoost achieved highest AUC (0.62; 95% CI 0.51–0.73) and F1 score (0.25) among all evaluated algorithm in predicting AFib	Small-sample size, median imputation, no external validation, and lack of advanced time-to-event analysis models	The model identifies AFib recurrence risks, and guides post-ablation management, with future potential via larger samples and validation

Study settings varied widely, including acute hospital care, ambulatory monitoring, and primary care-based risk screening. AF subtypes, where specified, were primarily paroxysmal or post-ablation recurrence.

### Overview of AI in AFib management

The application of AI in the primary healthcare setting has significantly improved patient outcomes and management of AFib. Wearable and wireless devices, such as smartwatches and biosensors, enable a user-friendly cardiac monitoring system that improves AFib detection. Concurrently, neural network-based models, including deep learning and convolutional neural networks, have demonstrated diagnostic accuracy surpassing that of experienced physicians. Machine learning algorithms, such as random forest (RF) and XGBoost (XGB), offer a promising approach to predicting arrhythmias among high-risk patients. The results highlight how the integration of AI within healthcare models is driving a transformative shift in AFib care and improving patient outcomes. Individual study characteristics are summarized in Table 2.

### Wireless/wearable technologies

Wireless and wearable technologies provide accessible, real-time AFib diagnostics with benefits in ambulatory settings. The SanketLife device showcased high sensitivity (98.15%) and specificity (100%) in diagnosing major cardiovascular conditions [11]. The wearable ECG wristband, integrated with a mobile AI app, demonstrated improved diagnostic accuracy across various postures (97.37%) and sensitivity (94.34%), offering a viable solution for detecting paroxysmal AF in home environments [12]. The wearable AI wristband was evaluated in ambulatory, asymptomatic individuals, primarily targeting paroxysmal AFib in a remote, user-operated setting. The BigThumb handheld ECG monitor, used by Huang et al., showed lower AFib recurrence detection rates post-ablation (64.2% vs. 78.9%, *p* = 0.0163) but achieved high diagnostic sensitivity (94.4%) and specificity (98.5%) [13]. The handheld AI-based ECG device was used for post-ablation AF recurrence monitoring in a structured outpatient cardiology setting, supporting high-risk follow-up care.

### Neural networks

Neural networks (NNs), particularly deep learning architectures such as CNNs and DNNs, have been applied in ECG analysis to enhance arrhythmia detection through automated pattern recognition [23]. Zhu et al. (2020) developed a CNN using 180,112 ECGs from over 70,000 patients for training and validation and tested it on 828 new ECGs. The model correctly diagnosed abnormalities in 658 (80%) cases, outperforming physicians with more than 12 years of experience, who averaged 621 (75%) correct diagnoses [14]. The model also outperformed physicians in identifying both single- and multi-label AFib cases, with a mean AUC-ROC score of 0.983 (95% CI 0.980–0.986), a sensitivity of 0.867 (0.849–0.885), and a specificity of 0.995 (0.994–0.996) [14]. Concurrently, Cai et al. (2020) introduced multi-ECGNet, incorporating ResNet, SE modules, and depthwise separable convolution. Trained on 24,106 ECGs and tested on 8,036, it achieved a micro-F1 score of 0.863 for AFib classification—exceeding cardiologists’ performance (0.780) [15]. Gruwez et al. developed a DNN to detect paroxysmal AF from sinus rhythm ECGs, training on 494,042 ECGs from 142,310 patients, with a sensitivity of (95% CI 79.0–82.3%) and specificity (95% CI 79.5–83.4%) [16].

### Machine learning

Machine learning (ML) models, such as random forest (RF), support vector machine (SVM), and eXtreme gradient boosting (XGB), have been applied to predict AFib and related risk factors using structured clinical and electronic medical record (EMR) data [24]. Kim et al. (2022) developed RF, SVM, and XGB models to predict atrial high-rate episodes (AHREs) before pacemaker implantation using data from 721 patients [17]. The ML models demonstrated higher AUROC values than logistic regression (RF: 0.742, SVM: 0.675, XGB: 0.745) and better calibration, as indicated by Brier scores (XGB: 0.021 vs. LR: 0.013) [17]. Kao et al. used RF, decision tree logistic regression (DTLR), and SVM to predict new-onset AFib in older adults based on medication, diagnostic, and laboratory data [20]. RF showed the best performance with an AUROC of 0.74 and a specificity of 98.7%. The AUROC and AUPRC derived from the RF algorithm were 0.74 and 0.89, respectively [20]. Gue et al. evaluated that among the machine learning models predicting atrial fibrillation as an adverse event, AdaBoost achieved the highest performance with an accuracy of 67.0%, F1 score of 0.25, and an AUC of 0.62 (95% CI 0.51–0.73), outperforming XGBoost, random forest, and neural network models across most metrics [19]. Ma et al. developed an explainable RF model using SHAP (SHapley Additive exPlanations) analysis to predict the recurrence of paroxysmal AFib after ablation, identifying top predictors such as CHA2DS2-VASc score, systolic blood pressure, and AFib duration [21]. Machine learning models, particularly AdaBoost, demonstrated potential utility in predicting atrial fibrillation (AF) as an adverse event, achieving the highest AUC (0.62; 95% CI 0.51–0.73) and F1 score (0.25) among all evaluated algorithms. These models incorporated key clinical variables, such as age, systolic blood pressure (SBP), CHA₂DS₂-VASc, and HAS-BLED scores, which were further validated using optimal thresholds derived from Youden index analysis [21, 24]. Hill et al. implemented the ML-based PULsE-AI algorithm in a UK primary care setting, identifying approximately 45,493 new AFib cases in a high-risk population of 3.3 million, with 50% sensitivity and 90% specificity [18].

## Discussion

### Comparisons across AI modalities

This review compares three primary AI technologies for AFib diagnostics: neural networks, wearable-integrated tools, and machine learning (ML) models, focusing on studies from 2020 to 2024, and includes head-to-head performance comparisons between the models and clinicians.

Wearable AI technologies, while slightly lower in diagnostic precision than NN, offered advantages in real-time ambulatory monitoring, with sensitivities above 94% [12]. Devices like the SanketLife wireless ECG biosensor and smartwatches demonstrated high usability and timely arrhythmia detection [11, 25, 26]. Yet, their reliance on patient compliance, single-lead ECGs, and testing in narrow populations limits broader clinical reliability. These models may reduce the time between arrhythmia onset and diagnosis, supporting more timely interventions in outpatient cardiology [13, 27]. Head-to-head comparisons with clinicians and validation across diverse settings are still needed [28].

Neural networks, especially convolutional and deep neural networks, demonstrated the highest diagnostic accuracy, often exceeding clinician performance in retrospective ECG analyses (80% vs. 75%) [14]. For instance, DNNs have shown promise in detecting paroxysmal AFib from sinus rhythm ECGs, which is helpful for post-visit screening or population-level risk assessment [16]. However, these models were trained on limited, homogeneous datasets and lacked prospective validation, raising concerns about generalizability [14, 15]. However, limitations, such as retrospective design, lack of multicenter data, and dataset imbalance, highlight the need for broader clinical validation and testing in real-world settings.

ML technologies showed relatively strong accuracy (AUROC 0.74–0.89) in identifying high-risk individuals in developing AFib, incorporating genetic and social factors from EMR [20]. However, most of these models were developed using retrospective data and lacked external validation, limiting their immediate clinical applicability. Overall, machine learning (ML) tools, when trained on large datasets, can aid clinicians in efficiently identifying arrhythmias while maintaining care quality [29]. ML strength lies in risk prediction and stratification, but like NNs, they were primarily retrospective and lacked external validation.

Together, these modalities offer complementary strengths: neural networks for high-accuracy ECG analysis, wearables for accessible real-time detection, and ML models for risk stratification, each with distinct limitations that require further validation and refinement.

### Clinical relevance

As artificial intelligence becomes increasingly integrated into cardiovascular diagnostics, understanding its practical applications is essential for effective clinical translation. Clinicians can consider integrating AI tools into AFib care, depending on the setting and subtype. Wearable AI devices are beneficial for detecting paroxysmal or asymptomatic AFib in ambulatory or postoperative monitoring [12, 16]. Neural networks can support ECG analysis in high-volume clinics, while machine learning models integrated into EHR systems may enhance preventative care by identifying high-risk individuals using genetic and social data [30]. Physicians should prioritize tools with external validation, transparent algorithms, and clear demographic reporting. Clinical use should be tailored to the societal context, as some models are better suited for detecting latent AFib via routine ECGs, while others leverage EMR data to identify incident AFib in older populations [31]. As evidence continues to evolve, cardiologists must remain informed about the strengths and limitations of each modality to ensure the responsible, equitable, and impactful implementation of patient care.

### Limitations

Although AI models demonstrated high diagnostic performance in controlled settings, this review is limited by the predominance of single-center, small-sample studies with minimal population diversity, often underrepresenting racial minorities and low-resource settings. For instance, Zhu et al. and Cai et al. relied on Chinese hospital data with limited geographic diversity, while Gruwez et al. used Belgian ECG datasets with unclear socioeconomic variation [14–16]. Most studies employed retrospective designs and had an unclear risk of bias in patient selection and follow-up timing, as indicated by the QUADAS-2 assessment, which limits their external validity and generalizability. This reduces external validation and can limit reliability and generalizability. Furthermore, performance metrics, such as AUROC, sensitivity, and specificity, were inconsistently reported, making cross-study comparisons challenging. No formal meta-analysis was performed due to heterogeneity in study design, AI model types, and reported outcome metrics. As a result, findings are presented descriptively without pooled estimates. Finally, the potential for publication bias remains, as studies with negative or null results may be underrepresented in the included literature. Additionally, rare or complex arrhythmias, such as asymptomatic paroxysmal AFib, may have reduced diagnostic accuracy across models. These models also rely on patient adherence, especially the wearable devices, for accurate detection of arrhythmias. None of the included studies assessed cost-effectiveness, which is critical for equitable implementation, particularly in under-resourced communities. This review also excluded studies written in non-English languages, which may have introduced language bias and limited the inclusion of relevant international research.

Ethical concerns remain unresolved, including patient data ownership, privacy, and liability—whether it falls on the clinician, AI developer, or healthcare institution. Regulatory ambiguity further complicates adoption: while the US Food and Drug Administration (FDA) has approved some AI-based cardiac tools under the Software as a Medical Device (SaMD) framework, ongoing model updates, reimbursement, and accountability remain underregulated [32]. The responsible integration of AI in AFib care will require not only technical performance but also transparency, inclusivity, and alignment with evolving legal and ethical standards.

### Future directions

Future research should focus on improving the reliability, scalability, and equity of AI models in AFib management. Advancing these tools can enhance diagnostic accuracy and timeliness while also improving healthcare system efficiency and patient outcomes. To ensure clinical applicability, studies should prioritize the detection of underrepresented and complex arrhythmias, aiming to strengthen model sensitivity and specificity across diverse populations. The development of interpretable AI models—using techniques such as SHAP values, saliency maps, or attention mechanisms—can provide clinicians with greater insight into algorithmic decision-making, fostering trust and adoption. Moreover, standardized reporting using AI-specific checklists, such as CONSORT-AI and SPIRIT-AI, is essential to ensure consistency in metrics, dataset transparency, and model disclosure [33]. Equity-focused algorithm design should include publicly available datasets annotated with race, sex, and socioeconomic metadata to support reproducible and inclusive model training. Extensive, multicenter, longitudinal studies involving diverse populations are also needed to evaluate long-term clinical outcomes and generalizability. Lastly, future research should assess how AI outputs are integrated into electronic health records (EHRs), influence clinician decision-making, and engage patients in their care workflows.

## Conclusion

This systematic review synthesizes current clinical evidence on AI technologies for atrial fibrillation (AFib) diagnostics, including neural networks, machine learning algorithms, and wearable-integrated models. While these models often outperform traditional methods in controlled settings, particularly in detecting asymptomatic or paroxysmal AFib, real-world application is limited by retrospective designs, small or homogeneous cohort groups, and limited external validation. The risk of bias, especially in patient selection and study flow, further limits generalizability. Nonetheless, AI models trained on ECGs or electronic health records offer significant potential for risk stratification, secondary screening, and recurrence monitoring, particularly in high-risk or resource-limited clinical environments. Wearable devices, in turn, enhance accessible remote and ambulatory monitoring. Clinicians should be informed of each modality’s use case and limitations, especially around interpretability and equity, and advocate for prospective, explainable, and workflow-aligned AI tools. When properly validated, AI integration into AFib care may reduce diagnostic delays, improve individualized management, and support a shift toward preventative, cost-effective cardiology. As the field advances, close collaboration between clinicians, data scientists, and regulatory bodies will be essential to ensure that AI technologies are safe, equitable, and truly transformative in AFib care.

## Data Availability

No datasets were generated or analysed during the current study.

## Abbreviations

CVD: Cardiovascular diseases
AFib: Atrial fibrillation
AI: Artificial intelligence
MRI: Magnetic resonance imaging
CTA: Computed tomography angiography
ECG: Electrocardiogram
ML: Machine learning
DL: Deep learning
PRISMA: Preferred reporting items for systematic reviews and meta-analyses
RF: Random forest
SVM: Support vector machines
SR: Sinus rhythm
XGBoost: EXtreme gradient boosting
SE: Squeeze-and-excitation
NN: Neural networks
CNN: Convolutional neural network
DNN: Deep neural network
PxAFib: Paroxysmal AFib
ResNet: Residual neural network
SHAP: SHapley additive exPlanations
SVM: Support vector machine
AUROC: Area under the receiver operating characteristic curve
AHREs: Atrial high-rate episodes
EHR: Electronic health record
DTLR: Decision tree logistic regression
AUPRC: The area under the precision–recall curve
AdaBoost: Adaptive boosting
WARCEF: Warfarin and aspirin in patients with heart failure and sinus rhythm
FDA: Food and drug administration
SaMD: Software as a medical device
HAS-BLED: Hypertension, abnormal renal/liver function, stroke, bleeding history or predisposition, labile inr, elderly, drugs/alcohol concomitantly
